# Abnormal myocardial perfusion in hypertrophic cardiomyopathy: preliminary findings of a cardiovascular MRI study

**DOI:** 10.1186/1532-429X-11-S1-P55

**Published:** 2009-01-28

**Authors:** James A White, Sarah Armstrong, Mohammed Al-Admawi, Sherryn Rambihar, Gerald Wisenberg, Ivonna Verschuur, Anna MacDonald, Cyndi Harper-Little, Aaron So, Ting-Yim Lee, Frank Prato, Terry Thomspon

**Affiliations:** 1 Dept. of Medicine and Lawson Health Research Institute and Robarts Research Institute, University of Western Ontario, London, ON Canada; 2 Dept. of Medicine, University of Western Ontario, London, ON Canada; 3 Robarts Research Institute, University of Western Ontario, London, ON Canada; 4 Lawson Health Research Institute, University of Western Ontario, London, ON Canada

**Keywords:** Myocardial Perfusion, Hypertrophic Cardiomyopathy, Myocardial Fibrosis, Perfusion Abnormality, Stress Perfusion

## Background

Reduced myocardial perfusion has been speculated as a potential mechanism for the development and/or propagation of myocardial fibrosis in hypertrophic cardiomyopathy (HCM). This study aims to evaluate the prevalence, distribution and extent of stress-induced perfusion abnormalities and their relationship to underlying fibrosis in patients with HCM using magnetic resonance imaging.

## Methods

15 patients with echocardiographically diagnosed HCM have been enrolled. Cine imaging, first-pass stress perfusion imaging using vasodilator stress (Dipyridamole), and delayed gadolinium enhancement imaging were performed. Stress hypoperfusion and delayed enhancement images were assessed both quantitatively and visually using a 16-segment model. Conversion of segmental visual scoring to % of LV by volume was achieved for both hypoperfusion (HP) and late enhancement (LE) using a standardized scoring system. For quantitative assessment prospectively defined cut-offs for LE and HP were used.

## Results

**Figure 1:**
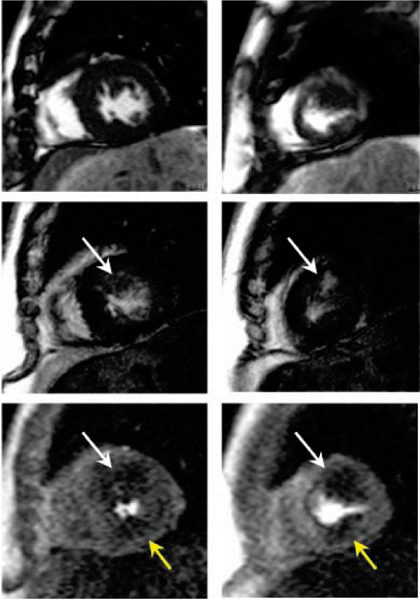
**Short axis cine** ***(top)*** **, delayed contrast** ***(middle row)*** **and stress perfusion** ***(bottom row)*** **images in a patient with apical hypertrophic cardiomyopathy**. Stress perfusion abnormalities seen corresponding to (white arrows) and distinct from (yellow arrows) established fibrosis.

Maximal wall thickness ranged from 13 to 22 mm (mean 17 ± 2.6 mm). Non-ischemic pattern LE was present in 70% of patients. Perfusion abnormalities were identified on stress perfusion images in 80% of patients using visual analysis and 87% of patients using quantitative analysis. Perfusion abnormalities were predominantly subendocardial, and were regionally associated with segments containing LE (p < 0.01). Mean percent HP and mean percent LE were 17 ± 8.4% and 10 ± 9.3%, respectively by visual estimation and 20.0 ± 12.1% and 14.0 ± 7.4%, respectively by quantitative assessment. Figure 1.

## Conclusion

These preliminary results suggest that patients with HCM have a high prevalence of stress-induced myocardial hypoperfusion as represented by reduced first-pass gadolinium enhancement during vasodilator stress. This hypoperfusion appears to extend beyond regions of established LE suggesting a potential contribution of ischemia in the development and/or propagation of myocardial fibrosis in patients with HCM.

